# 

**Published:** 1900-09-29

**Authors:** 


					THE HOSPITAL. "The standard of nighcSt purity." ?Lancet. September 29th, 1900.
CADBURY'S U If^ CADBURY'S
coooA )% m cocoa
NO DRUGS OR ALKALIES USED.
(
'ClAscotY Western Infirmar
NO. 731. Vol. XXVIII. [^Newspaper!] SATURDAY,
September 29, 1900.
"Subseription Torms.J TWOPENCE.

				

